# *Clostridium sordellii* as a Cause of Fatal Septic Shock in a Child with Hemolytic Uremic Syndrome

**DOI:** 10.1155/2014/237674

**Published:** 2014-05-07

**Authors:** Rebekah Beyers, Michael Baldwin, Sevilay Dalabih, Abdallah Dalabih

**Affiliations:** ^1^Department of Child Health, University of Missouri, 400 Keene Street, Columbia, MO 65201, USA; ^2^Department of Molecular Microbiology and Immunology, University of Missouri, Columbia, MO 65201, USA

## Abstract

*Clostridium sordellii* is a toxin producing ubiquitous gram-positive anaerobe, mainly associated with trauma, soft tissue skin infections, and gynecologic infection. We report a unique case of a new strain of *Clostridium sordellii* (not present in the Center for Disease Control (CDC) database) infection induced toxic shock syndrome in a previously healthy two-year-old male with colitis-related hemolytic uremic syndrome (HUS). The patient presented with dehydration, vomiting, and bloody diarrhea. He was transferred to the pediatric critical care unit (PICU) for initiation of peritoneal dialysis (PD). Due to increased edema and intolerance of PD, he was transitioned to hemodialysis through a femoral vascular catheter. He subsequently developed severe septic shock with persistent leukocytosis and hypotension, resulting in subsequent death. Stool culture confirmed Shiga toxin producing *Escherichia coli 0157:H7*. A blood culture was positively identified for *Clostridium sordellii*. *Clostridium sordelli* is rarely reported in children; to our knowledge this is the first case described in a pediatric patient with HUS.

## 1. Introduction

*Clostridium sordellii* is a toxin producing ubiquitous gram-positive rod anaerobe, mainly associated with trauma, soft tissue skin infections, and gynecologic infection [1]. It is known to cause a severe shock syndrome characterized by a leukemoid reaction and significant capillary leak [2]. It has a characteristically high mortality rate, approaching 70% in previously healthy individuals [3]. It is rarely described in children and rarely isolated from the blood. It has been previously reported to cause sepsis and omphalitis in children [4], but never in association with other infections. We describe the case of a toxic shock syndrome caused by a new strain of* Clostridium sordellii* in a previously healthy two-year-old male, with acute* Escherichia coli 0157:H7* positive hemolytic uremic syndrome.

## 2. Clinical Presentation

A previously healthy two-year-old male was admitted to the general pediatric floor with a two-day history of abdominal pain, vomiting, and diarrhea. The diarrhea was initially watery and became bloody at the time of admission. Vital signs and laboratory values on admission were normal except for white blood cells (WBCs) of 25.9 10^3^/*μ*L (83% neutrophils and 7% bands). His hemoglobin was 14.8 g/dL and platelets were 427 10^3^/*μ*L, and initial BUN and creatinine were 17 and 0.3 mg/dL, respectively. Abdominal ultrasound showed no abnormal findings.

The following day he had decreased urine output, elevated blood pressure, lower extremity and eyelid edema, and fever of 38.5°C. Vital signs and laboratory values at that time revealed tachycardia and elevated WBCs 34.6 10^3^/*μ*L (61% neutrophils and 20% bands); hemoglobin dropped to 10.8 g/dL and platelets to 43 10^3^/*μ*L. LDH level was 1319 unit/L and C3 and C4 were 76 and <9 mg/dL, respectively (normal values for a 2-year-old male are as follows: C3 = 80–170 mg/dL and C4 = 14–44 mg/dL). The peripheral smear showed toxic granulations and megakaryocytes and schistocytes. Abnormal electrolytes at that time were serum sodium of 131 mmol/L, potassium of 4.8 mmol/L, and elevated serum creatinine (0.9 mg/dL). Urine output had decreased from 3 cc/kg/hour to 1.3 cc/kg/hour. A presumptive diagnosis of hemolytic uremic syndrome (HUS) was made and he was transferred to the pediatric critical care unit (PCCU) for further management. Repeated stool studies and blood cultures were obtained upon transfer to the PCCU. A chest radiograph showed hazy bilateral airspace opacities and small right pleural effusion. Upon admission to the PCCU a peritoneal dialysis (PD) catheter (Covidien, Dublin) was inserted, and a 3 lumen (5 French/12 cm) central line (Cook Medical, IN) was placed in the left femoral vein. Next day in the PCCU the fever recurred and the peritoneal dialysis catheter became nonfunctional. The blood culture from the day before became positive for gram-positive rods, growing in anaerobic media. A new culture was repeated before starting antibiotics and the patient was started on cefepime and piperacillin-tazobactam for empiric coverage. At that time vancomycin was avoided due to impaired renal function. The stool culture and toxin testing from the day of admission was also reported positive for Shiga toxin producing* Escherichia coli 0157:H7*.

In the following few hours the patient became progressively oliguric, with increasing serum creatinine level to 1.6 mg/dL and persistent hypotension. Laboratory values at day 2 in the PCCU showed WBC 56.7 10^3^/*μ*L (29% neutrophils, 28% bands), Hgb 13.2 g/dL, and platelets of 26 10^3^/*μ*L. Due to altered mental status he was emergently intubated and after insertion of a right side 11.5 French femoral hemodialysis catheter hemodialysis was started. Due to the hypotension and acidosis (lactate level of 15 mmol/L) the patient was started on epinephrine and dopamine infusions (peak infusion rates of 15 mcg/kg/minute of dopamine and 0.1 mcg/kg/minute of epinephrine). He continued to be hemodynamically unstable, so a stress dose of hydrocortisone was administered. Clindamycin was added for empiric treatment of toxin production. Throughout the day, the patient continued to be hypotensive not responsive to fluid resuscitation or inotropic medications. Within 36 hours of admission to the PCCU hypotension persisted and toxic shock syndrome worsened; shortly after that, the patient arrested and was pronounced dead.

The repeated stool culture was also noted to be positive for* E. coli 0157:H7* and tested positive for Shiga toxin. Abdominal and pleural fluid cultures did not grow any organisms. Final blood culture identification was* Clostridium sordellii*. This identification was confirmed from 2 blood cultures 24 hours apart in the hospital laboratory and a plated sample was forwarded to an expert at the state laboratory. Repeated manual identification confirmed* Clostridium sordellii* (Table 1). When the samples of the bacteria were sent to the CDC, the center examined the samples and reported that this was a new strain of* Clostridium sordellii* not currently present in the CDC database.

## 3. Materials and Methods

### 3.1. Cell Cytotoxicity of Culture Supernatants

**Figure 1:**
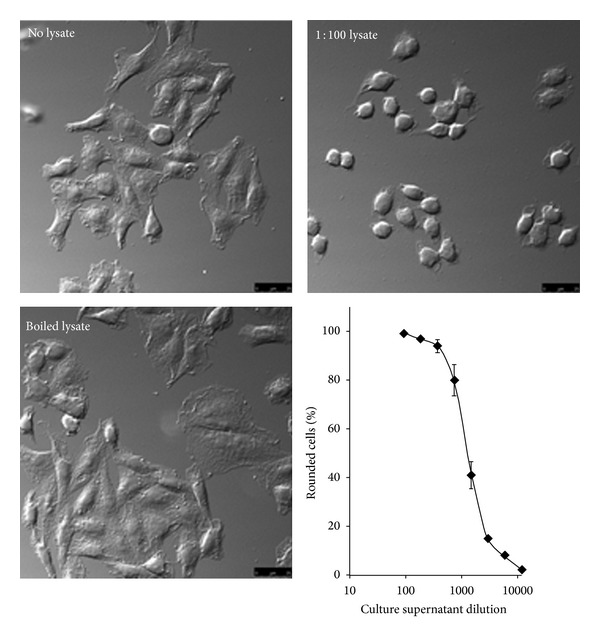
Supernatant cytotoxicity of* Clostridium sordellii* isolate. Culture supernatant (48 h) C. sordellii was incubated with an ∼50% confluent Vero cell monolayer in a 96-well plate in a total of 100 *μ*L per well and observed for 2 hr for cytopathic effects (CPE). Pictures shown are representative of a routine cell treatment with the indicated supernatant dilution, and cells were observed at a magnification of ×40. As a negative control, culture supernatant was heated to 90°C for 10 min and cooled prior to addition to cells. The degree of cytopathic effect was plotted against the relative dilution of the culture supernatant and is representative of eight independent trials.

Vero cells were either left untreated (control) or incubated with various dilutions of sterile culture filtrates in medium containing fetal cell serum (FCS) for 2 hours at 37°C. Morphological changes of intoxicated cells were directly analyzed in wells using an inverted microscope equipped with a DIC prism (Figure 1).

### 3.2. PCR Analysis of Sordellilysin Expression in* C. sordellii*

**Figure 2:**
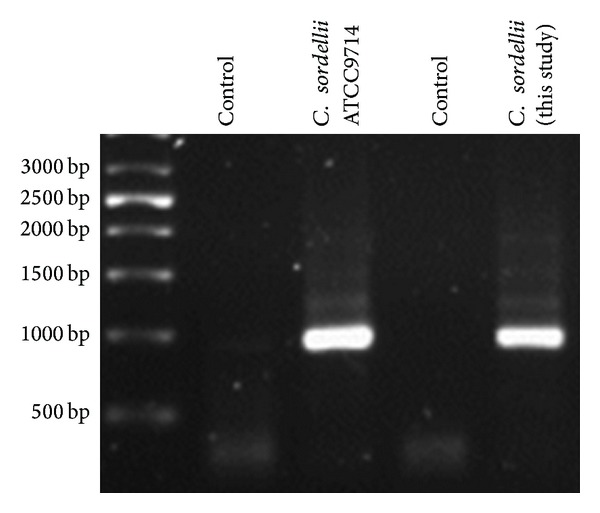
Analysis of sordellilysin in* Clostridium sordellii* isolates. Primers designed to amplify cholesterol dependent cytolysins (*cdc*) based on the reported sequence of perfringolysin O (*pfo*) were included in PCR reactions for each* C. sordellii* isolate. Amplification products were separated by 0.8% agarose gel electrophoresis and visualized via ethidium bromide staining. Lanes are designated by the strain names of each isolate in the figure.* C. sordellii* ATCC9714 was used as a positive control from sordellilysin expression.

Oligonucleotides (Integrated DNA Technologies Inc.) (Forward primer = 5′-GTACATATCCAGGAGCATTACAAC-3′; Reverse primer = 5′-CCACCATTCCCAAGCAAGACCTGT-3′) were designed to amplify sordellilysin based on the reported sequence of perfringolysin O [5]. PCR was performed using* Pfu* high fidelity polymerase (Agilent Genomics) and appropriate cycling conditions. Products were separated via agarose gel electrophoresis and compared with corresponding products from* C. sordellii* ATCC9714 (Figure 2).

### 3.3. Detection of Large Clostridial Toxins via Inhibitory Antibodies

Certain toxigenic isolates of* C. sordellii* produce toxins that are very similar to the toxins of* C. difficile*. The hemorrhagic toxin (toxin HT) of* C. sordellii* is very similar to toxin A whereas the lethal toxin (toxin LT) is very similar to toxin B. Antibodies against the* C. difficile* toxins neutralize the toxins HT and LT of* C. sordellii*, and antibodies against toxins HT and LT neutralize toxins A and B. To confirm the absence of HT or LT expression in our* C. sordellii* isolate a* C. difficile* Toxin/Antitoxin Kit (Techlab, VA) was used in conjunction with a Vero cell culture cytotoxicity assay in accordance with the manufacturer's instructions. In brief, serial dilutions of culture supernatants from* C. difficile* 630,* C sordellii* ATCC9714, and* C. sordellii* isolate XXX (our study) were mixed with either control or antitoxin antibodies prior to incubation within monolayers of Vero cells. Vero cells were monitored for up to 24 hr for cell rounding and visually scored. As expected, antitoxin antibodies effectively neutralized the toxin activity present in culture supernatants from* C. difficile* and* C. sordellii* ATCC9714, but not from* C. sordellii* isolate XXX.

## 4. Discussion

*Clostridium sordellii* is a spore forming gram-positive rod anaerobe found commonly in soil and the intestinal tract of mammals. Infections with* Clostridium sordellii* are mainly associated with trauma and medically induced abortions. It has been noted to be highly lethal in previously healthy individuals. It has been more recently described in normal term deliveries and heroin users [6]. There are over 40 different strains of* C. sordellii* and not all are toxin producing. Seven different exotoxins have been identified to be produced by* C. sordellii*, the two most virulent being lethal toxin and hemorrhagic toxin. These toxins are related to the large clostridial cytotoxin family, which affect cell signaling and are responsible for the characteristic leukemoid reaction and severe capillary leakage associated with* C. sordellii* infection [6]. Lethal toxin causes cell necrosis and edema secondary to increased vascular permeability. Hemorrhagic toxin has been noted to be directly cytotoxic* in vivo* studies [7]. The toxins have been known to directly depress cardiac output and systemic vascular resistance [6].

The initial absence of fever is a characteristic finding and often persists throughout the course of the illness. Severe symptoms of resistant hypotension and tachycardia follow quickly. Laboratory findings include leukocytosis, thrombocytosis, elevated hematocrit, and significant hypoalbuminemia. The leukocytosis is usually profound, often exceeding 75,000 and with a remarkable left shift, which we observed in our patient. Leukocytosis is also known to be a poor prognostic factor in HUS and highly associated with fatality in* C. sordellii* infection [6]. Pleural effusion and pulmonary edema are common and associated with capillary leakage and profound hypoalbuminemia [4].* Clostridium sordellii* infection not only carries a high mortality rate but is also associated with very rapid progression of the disease, with death occurring most commonly within 2–6 days of initial infection. In many reported cases, onset of severe hypotension and shock occurred within hours of symptom onset as in our case [4]. The rapid progression of the sepsis makes the infection difficult to identify and treat; our patient's blood culture identification was only available after the child had already died. Penicillin, tetracycline, and clindamycin have been tested* in vivo* for their efficacy in treatment. Clindamycin may have the added effect of decreasing toxin effect [5]. Treatment is mainly supportive in regard to hypotension and cardiovascular strain.

Although* C. sordellii* infection is relatively rare, there is suspicion that it is underreported, particularly in critically ill patients, due to its rapid progression and fastidious nature [6, 7]. A high index of suspicion, rapid initiation of treatment, and increased early identification would be key to decrease mortality associated with this organism. This case is unique in that* C. sordellii* infection has not been extensively described in children; isolation of* C. sordellii* from the blood is rare and to our knowledge has not been described in association with HUS. Although the exact source of* Clostridium sordellii* infection in this case is unknown, development of* C. sordellii* sepsis caused by translocation through the GI tract has been suspected in adult patients with comorbidities [6]. Hunley et al. had suggested that diarrhea associated HUS comprises a clinical entity which appears to predispose to a traumatic* C. septicum* infection, where acidic and anaerobic conditions in the diseased colon favor* C. septicum* invasion [8].

This could explain the mechanism of the suggested bacterial translocation in our patient. Clinicians should be aware of the possibility of clostridial infection in the presence of gastrointestinal pathology and hemodynamic instability, considering* Clostridia* and broadening the antibiotic coverage early on in the course might be lifesaving.

## Figures and Tables

**Table 1:** The list of the biochemical tests on the panel.

Substrates	Abbreviations	Organism reaction
p-Nitrophenyl-*β*-D-galactopyranoside	BGAL	Negative
p-Nitrophenyl-*α*-D-galactopyranoside	AGAL	Negative
bis-p-Nitrophenyl-phosphate	BPO4	Negative
p-Nitrophenyl-N-acetyl-*β*-D-glucosaminide	NGLU	Negative
p-Nitrophenyl-*α*-D-glucopyranoside	AGL	**Positive**
o-Nitrophenyl-*β*-D-glucopyranoside	BGL	Negative
p-Nitrophenyl-phosphate	PO4	Negative
p-Nitrophenyl-*α*-L-fucopyranoside	AFU	**Positive**
p-Nitrophenyl-*α*-D-mannopyranoside	MNP	Negative
L-Leucine-*β*-naphthylamide	LEU	Negative
DL-Methionine-*β*-naphthylamide	MET	Negative
L-Lysine-*β*-naphthylamide (alkaline)	LYB	Negative

Substrates	Abbreviations	Negative

L-Lysine-*β*-naphthylamide (acid)	LYA	Negative
Glycylglycine-*β*-naphthylamide	GGLY	Negative
Glycine-*β*-naphthylamide	GLY	Negative
L-Proline-*β*-naphthylamide	PRO	**Positive**
L-Arginine-*β*-naphthylamide	ARG	Negative
L-Pyrrolidonyl-*β*-naphthylamide	PYR	Negative
L-Tryptophan-*β*-naphthylamide	TRY	Negative
3-Indoxyl phosphate	IDX	Negative
Trehalose	TRE	Negative
Urea	URE	**Positive**
Indole	IND	**Positive**
Nitrate	NIT	Negative

This set of biochemical reactions was compared to the MicroScan Database for *Clostridia* species. The result for this set of positive and negative biochemical reactions was consistent with a 99.99% probability of *Clostridium sordellii*.

## Abbreviations

HR: Heart rate
WBCs: White blood cells
PCCU: Pediatric critical care unit
PD: Peritoneal dialysis
CDC: Center for Disease Control
FCS: Fetal cell serum
CPE: Cytopathic effects
HUS: Hemolytic uremic syndrome.
